# Inadvertent post-expiry COVID-19 vaccination in 282 seniors: Safety, efficacy, and implications for extended off-label stability data

**DOI:** 10.1016/j.puhip.2026.100802

**Published:** 2026-05-10

**Authors:** Henrik Klasson

**Affiliations:** Vårdcentralen Getingen, Lund, Sweden

**Keywords:** COVID-19 vaccines, mRNA vaccines, Expired vaccines, Vaccine stability, Public health preparedness

## Abstract

**Objectives:**

To describe safety and clinical outcomes after inadvertent post-expiry administration of Pfizer-BioNTech SARS-CoV-2 vaccine to adults aged ≥80 years in Swedish primary care; indicate what can (and cannot) be inferred about efficacy from real-world follow-up; and identify system needs for clearer expiry labelling and producer-led, longer-horizon post-expiry evidence. The study was not designed to estimate vaccine effectiveness.

**Study design:**

Service evaluation; observational single-arm cohort (ambispective).

**Methods:**

After discovery that 282 recipients had received doses 3–5 months past the labelled expiry, 280 survivors were informed and offered in-date revaccination within ∼4 weeks. Clinicians recorded revaccination uptake, adverse events exceeding expected post-mRNA symptoms after expired and (if applicable) in-date doses, and any confirmed/strongly suspected COVID-19 prior to revaccination; deaths were attributed via records. A change in manufacturer presentation of shortened/altered expiry encountered locally at point of care was documented.

**Results:**

208/280 accepted revaccination; 72/280 declined. Two deaths occurred from age-related/pre-existing illness (not vaccination-related). No serious or unexpected adverse events were recorded after expired or in-date doses. A small number reported mild, self-limited illness compatible with COVID-19; there were no hospitalisations and no laboratory-confirmed cases.

**Conclusions:**

In this cohort of older seniors, inadvertent post-expiry vaccination was followed by no safety signal and no COVID-19 hospitalisations; effectiveness cannot be quantified from this design. Given recurring human error and cold-chain stress, clearer expiry labelling and transparent, real-life post-expiry stability/efficacy data (weeks–months; including temperature-excursion profiles) are needed to support patient safety, reduce wastage, and strengthen preparedness.

## 1 Introduction

In routine operations at a primary care clinic in southern Sweden, 282 individuals aged ≥80 years received a SARS-CoV-2 vaccine booster that was past the stated expiry date (November 27th, 2024). The vaccination occurred between March and May 2025. The deviation came to light during an internal stock check post-vaccination. The affected cohort—older seniors and with or without other risk factors—prompted immediate disclosure and mitigation. Two (2) deaths occurred during follow-up and were attributed to age-related or pre-existing illness, not vaccination, leaving 280 patients to be offered revaccination. Of these, 208 patients accepted revaccination within ∼4 weeks; 72 declined. Revaccination was completed by end-July.

Prior reports show that post-expiry administration does occur and is likely under-reported. In U.S. surveillance of expired injectable influenza vaccines, adverse events were rare and non-serious, with no excess risk signal; efficacy was not assessed [1]. In Brazil, alleged post-expiry COVID-19 administration prompted guidance to revaccinate and highlighted the absence of publicly available, longer-horizon stability/efficacy data [2] Expiry-related wastage remains a persistent systems issue globally [3].

Two considerations motivated a structured follow-up. First, patient safety: whether expired mRNA vaccines had any unusual reactogenicity or toxicity. Second, effectiveness: whether expired product conferred any meaningful protection pending revaccination. This report summarizes outcomes and draws policy implications for manufacturer labelling and post-expiry evidence.

## 2 Methods

All 280 patients were contacted by letter and/or telephone, informed of the incident, and offered revaccination with an in-date Pfizer-BioNTech dose within ∼4 weeks. Follow-up occurred through standard clinical contacts and quality assurance framework Information of the two deaths and their clinical attribution was based on medical records. No experimental procedures, biological sampling, or identifiable data extraction for research were performed.

From the 208 who accepted, data was collected at point of revaccination through a health provider questionnaire. The health providers questionnaire did ask of the number of previous COVID-19 vaccinations, but did not ask specific questions on previous covid-illness, leaving this up to auto-anamesis at time of vaccination.

Monthly incidence for COVID-19 in the age cohort ≥80 years was obtained from The Public Health Agency of Sweden for the period March-July.

Direct communication with vaccine producers and The Swedish Medical Products Agency was made, requesting off-label data on stability and efficacy which was swiftly provided. However, data only existed for two (2) days post expiry.

## 3 Results

### 3.1 The revaccination cohort

Of 282 patients who received an expired dose, two (2) had died during follow-up. Clinical review attributed death to age-related or pre-existing conditions without temporal or causal link to vaccination. Of the remaining 280 patients offered revaccination, 208 accepted (127 women, 81 men); 72 declined for undocumented reasons. As reasons for declining revaccination were not collected in a structured manner it cannot be characterised beyond non-uptake.

No serious or unexpected adverse events were recorded after either the expired or in-date doses or the revaccination. Occasional patients gave auto-anamnesis on adverse events associated with expected post-mRNA vaccination symptoms after the expired dose. A few patients gave auto-anamnesis of having had symptoms of a cold since vaccination, compatible with mild COVID-19. No testing had been done to assess the origin of symptoms. There was no auto-anamnesis on confirmed COVID-19 episodes after the expired dose and prior to revaccination, nor any Patient Discharge Summaries from national hospitals. Thus, there was no airway infection requiring hospitalisation in the cohort.

### 3.2 National incidence of COVID-19 infection

The Public Health Agency of Sweden provided incidence of COVID-19 for the period March-July for the age cohort of ≥80 years. Regional incidence ranged between 9 and 22 cases/month of laboratory-confirmed COVID-19 per capita, (mean and median of 15 per capita). The national incidence was higher, with a range of 22-40 cases/month during the five months (mean and median of 30 cases/month). These surveillance figures are provided solely to contextualise background transmission during the period. Because our cohort was not systematically tested and mild/asymptomatic infections may not have been detected, any comparison with regional/national incidence is prone to substantial under-ascertainment and can not be interpreted as evidence of vaccine effectiveness.

Applied mechanically, these rates would correspond to roughly 4–5 laboratory-confirmed cases per month in our small cohort of 282 patients at the regional level, and twice as many given the national incidence during the time-frame. However, because case ascertainment in our cohort was passive and on an “as need arise”-basis, this mathematical back-of-the-envelope conversion is highly uncertain and is presented only for context.

### 3.3 Ambiguous labelling at point of care

**Fig. 1:**
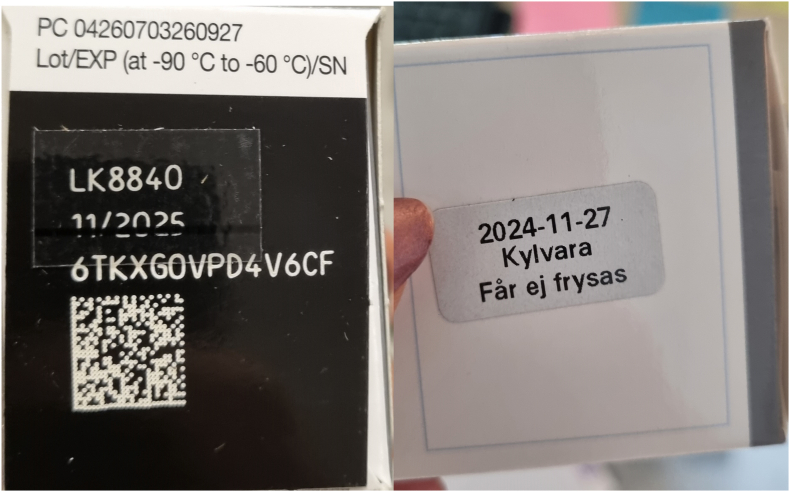
The two sides of the vaccine package, the left referring to the expiry date for the frozen (−80 °C) vaccine, the right referring to the thawed vaccine, after which expiry date is shortened compared to the frozen vaccine. This illustrates why a single point-of-use ‘Discard after’ date/time would reduce ambiguity.

The packaging has one expiry date for the frozen mRNA-vaccine printed on one side, the thawed vaccine (shortened) expiry date printed on the other (see Fig. 1). This opens for ambiguity at point-of-use.

## 4 Discussion

Human error will occur. Even in well-run services, latent system vulnerabilities—stock rotation, visual similarity of vials, multiple expiry schemes, and staffing pressures—can culminate in mistakes. The responsible response is transparency, prompt mitigation (revaccination), and learning. That no hospitalisation was seen in the cohort we attribute to providence.

**Labelling matters at the point-of-care.** We observed a change in the way altered/shortened expiry was presented after the event. Clearer, more salient expiry communication (e.g., unambiguous over-stickers, such as single final ‘Discard after’ date/time at point of care, applied as an over-sticker that supersedes all other expiry information once thawed. Ideally, this should be paired with machine-readable coding (barcode/QR) that vaccination software can flag as in-date/out-of-date) can be a powerful last-mile safeguard.

**Not a blame exercise—but a call for producer-led evidence.** This report exposes a systemic gap: publicly accessible off-label stability and efficacy data for vaccines beyond the labelled expiry is extremely limited—only a couple of days beyond expiry date. However, it is rarely so that a substance turns foul from one moment to the next. Natura non facit saltus—nature does not make jumps; i.e. biologics degrade along a continuum. It is not a phase-transition, it is a decay curve. More research is needed and manufacturers hold the proprietary knowledge, analytical platforms, and resources to generate longer-horizon data (weeks to months), both for safety (e.g., degradants/toxicity profiles) and for functional efficacy (e.g., potency/neutralisation correlates).

**Necessity of more off-label data studies**. Bhusal et al. show how on-site realities in Nepal—including intermittent power, equipment failure, and last-mile delays—drive vaccine discard through cold-chain breaks [3]. These conditions argue for producer-led off-label studies that model real-world temperature excursions and time-beyond-expiry so guidance reflects actual field risk rather than ideal storage alone.

The importance of more and better off-label data cannot be overstated. In disrupted supply chains—whether due to conflict at Europe's borders, strikes, extreme weather or pandemics—stock rotation lags and cold-chain contingencies are stressed; evidence-based guidance on time-beyond-expiry and temperature excursions would prevent waste while maintaining safety. Equitably, distant regions with limited cooling capacity could use validated stability margins to reduce the discard of otherwise functional doses. And for rational policy, decision-makers should not infer from isolated field mishaps but rely on transparent, producer-generated data to set conservative yet realistic rules for emergency use. If such data are not provided voluntarily, legislation will be required.

Our outcomes—no unexpected safety signal, no hospitalisations, and unknown short-term protection—do not justify expired vaccine use. They do, however, underscore that an evidence gap persists precisely where clinical teams need clarity most.

### 4.1 Limitations

This was a service evaluation, not a trial. We lacked systematic testing and immunogenicity measures. Instead, we rely on clinical data and self-assessment at revaccination. As with prior analyses of expired vaccines, our evaluation cannot quantify efficacy and should not be interpreted as establishing protective effectiveness beyond labelled expiry.

We could not analyse the reason for declining reactivation among the 72 patients, as this information was not available. Thus it is speculative if the decision was rooted in concern about additional doses, perceived adequacy of the expired dose, decreased trust in the medical establishment, or other reasons. Future incident-response protocols should include a brief, standardised ‘reason for decline’ field to inform targeted communication and logistics.

### 4.2 Conclusion

This older cohort experienced no serious harm following administration of expired vaccine doses and no COVID-19 infection requiring hospitalisation occurred. The event should not recur—but if it does, clinicians need better guidance. We urge vaccine manufacturers to lead on extended, transparent post-expiry stability and efficacy studies, so-called “off-label data”. Weeks and months—not days—should define the horizon of preparedness-relevant evidence. Extended, public post-expiry evidence and clearer expiry labelling are actionable levers that improve patient safety, reduce wastage, and strengthen global health-system preparedness.

## Ethical statement

This work was conducted as clinical quality assurance after an unintended deviation in routine care; use of vaccine passed expiry date. No experimental procedures were performed and no identifiable patient data were reported. Per institutional policy, quality-assurance evaluations of completed routine care do not require REC approval.

Given the rarity of published reports or filed-notes from post-expiration date use of vaccines and its efficacy and safety, the experience is shared here.

## Declaration of generative AI and AI-assisted technologies

During the preparation of this work the author used ChatGPT-5 (OpenAI) in order to condense and copy-edit text, improve readability and structure, and draft alternative phrasings where needed. After using this tool, the author reviewed and edited the content as needed and takes full responsibility for the content of the published article. No AI tools were used for data collection, analysis, interpretation, or drawing scientific conclusions.

## Funding

All costs for patient contact and revaccination logistics were covered by the primary care clinic where the author worked.

## Declaration of competing interest

⌧ The authors declare that they have no known competing financial interests or personal relationships that could have appeared to influence the work reported in this paper.

⌧ The author is an Editorial Board Member/Editor-in-Chief/Associate Editor/Guest Editor for this journal and was not involved in the editorial review or the decision to publish this article.

⌧ The authors declare the following financial interests/personal relationships which may be considered as potential competing interests: **None**.

## Data Availability

Anonymised aggregate data is available from the author upon reasonable request.
